# Full-Length Sequence of Mouse Acupuncture-Induced 1-L (*Aig1l*) Gene Including Its Transcriptional Start Site

**DOI:** 10.1093/ecam/nep121

**Published:** 2011-02-17

**Authors:** Mika Ohta, Aki Sugano, Shuji Goto, Surini Yusoff, Yushi Hirota, Kotaro Funakoshi, Kenji Miura, Eiichi Maeda, Nobuo Takaoka, Nobuko Sato, Hiroshi Ishizuka, Naoki Arizono, Hisahide Nishio, Yutaka Takaoka

**Affiliations:** ^1^Laboratory for Applied Genome Science and Bioinformatics, Clinical Genome Informatics Centre, Kobe University Graduate School of Medicine, Kobe 650-0017, Japan; ^2^Department of Biochemistry, Iwate Medical University School of Dentistry, Morioka 020-8505, Japan; ^3^Department of Acupuncture Informatics, Goto College of Medical Arts and Sciences, Tokyo 143-0016, Japan; ^4^Department of Genetic Epidemiology, Kobe University Graduate School of Medicine, Kobe 650-0017, Japan; ^5^Department of Clinical Molecular Medicine, Kobe University Graduate School of Medicine, Kobe 650-0017, Japan; ^6^Graduate School of Sciences, Kyushu University, Fukuoka 812-8581, Japan; ^7^Department of Anatomy, Tokushima University School of Dentistry, Tokushima 770-8504, Japan; ^8^Department of Medical Zoology, Kyoto Prefectural University of Medicine, Kyoto 602-8566, Japan

## Abstract

We have been investigating the molecular efficacy of electroacupuncture (EA), which is one type of acupuncture therapy. In our previous molecular biological study of acupuncture, we found an EA-induced gene, named acupuncture-induced 1-L (*Aig1l*), in mouse skeletal muscle. The aims of this study consisted of identification of the full-length cDNA sequence of *Aig1l* including the transcriptional start site, determination of the tissue distribution of *Aig1l* and analysis of the effect of EA on *Aig1l* gene expression. We determined the complete cDNA sequence including the transcriptional start site via cDNA cloning with the cap site hunting method. We then analyzed the tissue distribution of *Aig1l* by means of northern blot analysis and real-time quantitative polymerase chain reaction. We used the semiquantitative reverse transcriptase-polymerase chain reaction to examine the effect of EA on *Aig1l* gene expression. Our results showed that the complete cDNA sequence of *Aig1l* was 6073 bp long, and the putative protein consisted of 962 amino acids. All seven tissues that we analyzed expressed the *Aig1l* gene. In skeletal muscle, EA induced expression of the *Aig1l* gene, with high expression observed after 3 hours of EA. Our findings thus suggest that the *Aig1l* gene may play a key role in the molecular mechanisms of EA efficacy.

## 1. Introduction

Traditional Eastern medicine, such as acupuncture, moxibustion and Chinese herbal medicine, originated in ancient China and has developed unique forms in East Asian countries (mainly Japan, China and Korea). These practices are called traditional Japanese medicine (*Kampo*), Chinese medicine and traditional Korean medicine [1]. Electroacupuncture (EA) is a well-known acupuncture technique used in East Asia. EA utilizes stimulation of acupuncture needles with a low-frequency microcurrent [2].

Comprehensive analysis of changes in gene expression is called transcriptome analysis, which has provided a number of findings to medical science. cDNA microarray and GeneChip array methods allow such comprehensive gene expression analysis [3, 4]. Certain research groups have reported investigations of gene expression in EA-treated animals in which they used cDNA microarrays. Kim et al. [5] reported that 30 minutes of EA applied every 16 hours for 2 days induced a change in expression of genes related to natural killer cell activity in the spleen. Ko et al. [6] showed that gene expression of mitogen-activated protein kinase II (MAPK II), Fas-AP and LIM increased in spinal cord after 3 weeks of daily application of EA for 30 minutes. Chae et al. [7] reported research on acupuncture-treated humans that they analyzed by using a cDNA microarray; after correlating gene expression with responses to acupuncture stimulation, they found individual differences in acupuncture analgesia.

We have also analyzed the effect of EA at the gene transcriptional level [2]. In that study, we found that EA stimulation caused myostatin gene suppression leading to satellite cell proliferation in skeletal muscle. During that study, we discovered a previously unknown gene that was induced by EA in mouse skeletal muscle and named it *Aig1l*. We found high expression of this gene after EA stimulation in mouse skeletal muscle. This finding agrees with clinical observations that the effect of acupuncture appears some time after stimulation, and it led us to further analysis of this gene.

In the study described here, we used cDNA cloning with the cap site hunting method [8] to determine the full-length cDNA sequence of *Aig1l*, the full-length sequence consisting of the transcriptional start site to the poly(A) tail. Complete full-length cDNA sequencing is important for functional analysis of the gene; knowing the transcriptional start site makes it possible to evaluate the transcriptional regulatory domain by bioinformatics analyses. Indeed, we used bioinformatics to analyze the transcriptional regulatory domain of *Aig1l*. We also analyzed the full-length cDNA sequence by bioinformatics analysis and detected functional domains of *Aig1l*. Finally, we examined *Aig1l* expression in each tissue by using northern blot analysis and real-time quantitative polymerase chain reaction (real-time PCR). For the relation between the *Aig1l* gene and EA, we investigated *Aig1l* expression via semiquantitative reverse transcriptase polymerase chain reaction (RT-PCR) at different times after EA stimulation of muscle.

## 2. Methods

### 2.1. Animals and EA Conditions

Inbred C57BL/6 male mice, 8 weeks old, were purchased from Charles River Laboratories (Yokohama, Japan). For EA stimulation, we used stainless-steel acupuncture needles (40 mm long and 0.16 mm in diameter; Seirin, Shizuoka, Japan). We inserted the needles into five anesthetized mice to a depth of 5–7 mm and then stimulated the needles with an electrical stimulator (Kyushu Ryoudoraku, Fukuoka, Japan). Hindleg muscles of mice received EA stimulation at points corresponding to the acupoints BL36 and BL59 (for details, see our web site: http://bionano.med.kobe-u.ac.jp/adss/) for 15 minutes with 1.2 Hz repetitions, according to our previous study [2]. We similarly anesthetized a control group of five mice but did not treat them with EA.

This research was performed according to the Standards Relating to the Care and Management, and so forth. of Experimental Animals (Ministry of the Environment, Tokyo, Japan) [9]. This study was approved by the Committee for Safe Handling of Living Modified Organisms of Kobe University (Permission number 17–21) and was carried out according to the guidelines of the Committee.

### 2.2. RNA Extraction and cDNA Synthesis

We extracted total RNA from various tissues (brain, skeletal muscle, heart, lung, spleen, liver and kidney) obtained from non-EA-treated mice by using the TRIzol reagent (Invitrogen, Carlsbad, CA, USA) method.

To extract total RNA, we prepared skeletal muscles (gastrocnemius, soleus, biceps femoris and gluteus) from EA-treated mice at the time points of immediately after EA (0 hours) and then 1, 3 and 24 hours after EA (*n* = 5 for each time point). We chose these time points on the basis of our clinical experience and observations during acupuncture treatment, which we classified into two groups: “rapid effect" for immediately (0 hours) and 1 and 3 hours after EA, and “late effect" for 24 hours after EA. We similarly extracted total RNA from skeletal muscles from the control group. For each PCR analysis, we reverse transcribed total RNA (5 *μ*g) into cDNA by using the SuperScript First-Strand Synthesis System for RT-PCR (Invitrogen), according to the manufacturer's instructions.

### 2.3. Northern Blot Analysis

We subjected the denaturated total RNA (1 *μ*g) to electrophoresis on 1.2% agarose/2% formaldehyde gel and then transferred the samples to a positively charged nylon membrane. To generate anti-sense RNA probes, we cloned a 509 bp of *Aig1l* cDNA fragment (nucleotide positions 3720–4228) derived from the PCR amplification by using the TOPO TA cloning kit (Invitrogen). We prepared digoxigenin-labeled RNA probes with DIG RNA Labeling Mix (Roche Diagnostics GmbH, Mannheim, Germany). The membrane was hybridized with DIG-labeled RNA probes just described in DIG Easy Hyb (Roche Diagnostics) at 68°C overnight. Then, excess probe was washed away: 2× SSC/0.1% SDS was used twice at RT, and then 0.1× SSC/0.1% SDS was used twice at 68°C. Hybridized DIG-labeled RNA was detected by means of alkaline phosphatase-conjugated anti-DIG antibody (Roche Diagnostics), after which CSPD Substrate was added and the membrane was exposed to X-ray films to obtain signals. The membrane was rehybridized with the DIG-labeled mouse glyceraldehyde-3-phosphate dehydrogenase (G3PDH) RNA probe (Genostaff, Tokyo, Japan) as an internal control.

### 2.4. Real-Time Quantitative PCR and Semiquantitative RT-PCR

Real-time quantitative PCR was performed by using SYBR Green I and a LightCycler (Roche Diagnostics), according to the manufacturer's instructions. The reaction mixture consisted of 2 *μ*l of FastStart DNA Master SYBR Green I (Roche Diagnostics), 2 mM MgCl_2_, 2 *μ*l of cDNA and each primer at 10 pmol, plus water to a final volume of 20 *μ*l. The PCR conditions were 95°C for 10 minutes followed by 40 cycles in three steps: 95°C for 15 s, 58°C for 10 s and 72°C for 25 s and then 60°C for 10 s. The primers used for real-time PCR were as follows: for *Aig1l*, sense: 5′-TTGAAGCCAGCTCTTTGGAG-3′ and antisense: 5′-TTTGCCTACGGTTCCTGAAG-3′; for G3PDH, which served as an internal control [10], sense: 5′-GGAAAGCTGTGGCGTGATG-3′ and antisense: 5′-CTGTTGCTGTAGCCGTATTC-3′.

For semiquantitative RT-PCR, for which we used a thermal cycler (GeneAmp PCR System 9700; Applied Biosystems, Foster City, CA, USA), the reaction mixture (20 *μ*l) contained 5 pmol primers, 0.4 unit AmpliTaq Gold DNA polymerase (Perkin-Elmer/Cetus, Tokyo, Japan), 1× PCR buffer and 200 *μ*l/l dNTPs. We analyzed the *Aig1l* gene expression pattern after the EA stimuli by means of RT-PCR with primers as follows: sense: 5′-ACTGGGATACACTCGTGAGC-3′; antisense: 5′-GACACAGGAAGGTCACCACCA-3′. Primers of G3PDH for RT-PCR were the same as the ones used for real-time PCR. We then performed 28 cycles of amplification (one cycle: denaturation at 94°C for 60 s, annealing at 55°C for 60 s and extension at 72°C for 60 s), and we analyzed 449 bp PCR products from *Aig1l* by using 1.5% agarose gel electrophoresis. After electrophoresis, we determined the densitometric values of these PCR bands by the same procedure that we reported in our previous study [2], using the ImageJ program (Wayne Rasband, National Institutes of Health, Bethesda, MD, USA).

### 2.5. Full-Length cDNA Sequencing and Bioinformatics Analysis

We amplified a partial sequence of *Aig1l* by use of two primers—forward, 5′-ACTGGGATACACTCGTGAGC-3′, and reverse, 5′-GACACAGGAAGGTCACCACCA-3′—as probes. The *Aig1l* probe served to screen the mouse brain cDNA library (Stratagene, La Jolla, CA, USA). We screened with the labeled probe by means of the AlkPhos Direct system (Amersham Biosciences Corp., Piscataway, NJ, USA), according to the manufacturer's protocol. We isolated and purified positive clones. We then performed automated DNA sequencing analysis by using the ABI Prism 377 with the BigDye Terminator Cycle Sequencing Reaction Kit (Applied Biosystems). We confirmed sequencing results with both strands.

**Figure 1:**
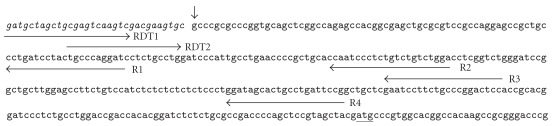
PCR primer sets for the cap site hunting method analysis of the *Aig1l* gene. The cap site hunting method allowed detection of the transcriptional start site of the gene. Horizontal arrows indicate positions of the primer pairs. In the first PCR, we prepared primer sets for the brain (RDT1 and R4) and skeletal muscle (RDT1 and R3). In the nested PCR, we prepared primer sets for the brain (RDT2 and R2) and skeletal muscle (RDT2 and R1). The vertical arrow points to the transcriptional start site. Underlining shows the start codon atg. Italic letters designate the synthetic rOligo sequence ligated to the mRNA.

We determined the transcriptional start site of the *Aig1l* gene by using the cap site hunting method with mouse skeletal muscle and brain cap site cDNA (Nippon Gene, Toyama, Japan), according to the manufacturer's instructions. The procedure for synthesis of cap site cDNA was reported previously [8] as follows: we removed the cap structure of mRNA with tobacco acid pyrophosphatase. We then ligated a synthetic oligoribonucleotide to the decapped mRNAs with T4 RNA ligase. We converted the ligated mRNA to cDNA by using reverse transcriptase, with oligo(dT) as a primer. In this study, we first performed PCR with the cap site cDNA and then performed nested PCR.  Figure 1 shows the primer sets for those PCRs. We amplified samples of the first PCR for 35 cycles under the following conditions: denaturation for 20 s at 95°C, annealing for 20 s at 60°C and extension for 90 s at 72°C. We used aliquots of the first PCR reaction as the template in the nested PCR reaction, performed under the same conditions. We then excised PCR products from the low-melting-temperature 1.5% agarose gel, inserted them into the TA vector, and sequenced them. After we sequenced the full-length cDNA, we performed bioinformatics analysis of *Aig1l* by using the Pfam database (http://pfam.sanger.ac.uk/). Then, to analyze the transcriptional control regions of *Aig1l* genes (upstream from the ascertained transcriptional starting point), we used SHAFT (https://suzume.med.kobe-u.ac.jp/SHAFT/), an automated application of the search and choice analyses for transcription factors that we previously developed [2].

## 3. Results

### 3.1. Cloning of the Full-Length cDNA Sequence of *Aig1l* and Bioinformatics Analysis of the Gene

**Figure 2:**
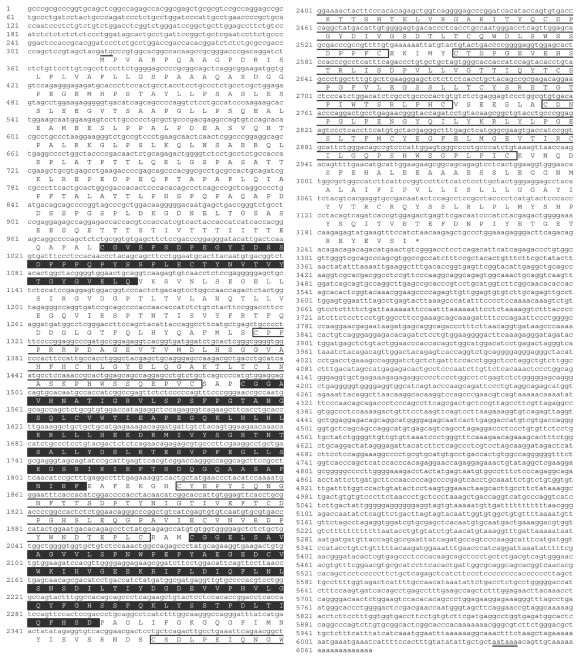
Nucleotide and deduced amino acid sequences of the *Aig1l* gene. The lowercase upper letters correspond to the nucleotide sequence. The capital letters refer to the deduced amino acid sequence. The numbers at the ends of the lines indicate nucleotide positions. Underlining shows the initiation codon atg. The closed boxes with white letters designate the CUB domain; the open boxes with black letters indicate the Sushi domain. An asterisk indicates the stop codon (taa). A double underline identifies the poly(A) signal sequence.

We determined the full-length cDNA sequence of the *Aig1l* gene via cDNA cloning and use of the mouse brain cDNA library, because the brain showed high expression of the gene. We ascertained the transcriptional starting point of the gene by means of the cap site hunting method with cap site cDNAs from mouse brain and from mouse skeletal muscle, because the transcriptional start site is not included in the usual cDNA libraries. We obtained the same transcriptional start site from cap site cDNA of both mouse brain and muscle. After we determined the complete cDNA sequence of *Aig1l*, we registered it with the GenBank genome database (GenBank accession no. DQ167195). The full-length cDNA sequence was 6073 bp long, and the putative protein consisted of 962 amino acids (Figure 2). Bioinformatics analysis performed via the Pfam database indicated that *Aig1l* includes CUB and Sushi domains. The transcriptional control region, near the transcriptional start site, of the *Aig1l* gene has Sp1, CP2 and MZF1 binding motifs that we detected by using the SHAFT program.

### 3.2. Tissue Distribution of the *Aig1l* Gene and Characteristics of Gene Expression Induced by EA

**Figure 3:**
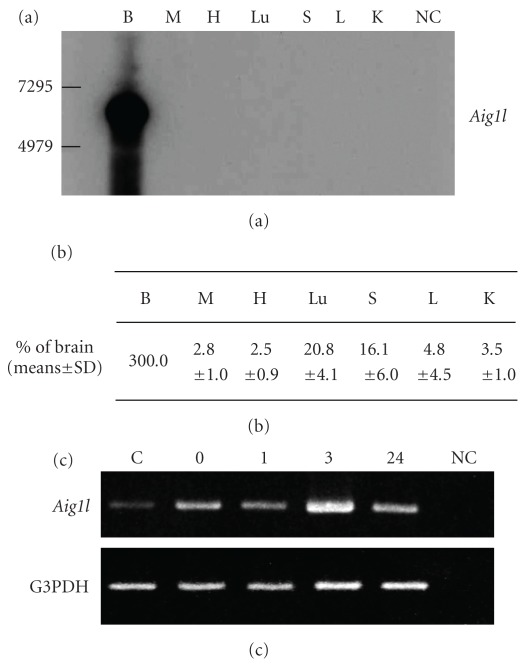
Tissue distribution and effect of EA on *Aig1l* gene expression. (a) Tissue distribution of *Aig1l* as detected by northern blot analysis in the normal sensitivity mode. (b) Tissue distribution of *Aig1l* as detected by real-time quantitative PCR analysis (*n* = 5). (c) Semiquantitative RT-PCR analysis of *Aig1l* gene expression in skeletal muscle after EA. Lanes are as follows: C: no EA stimulation (control); 0: EA-treated sample just after stimulation; 1, 3 and 24: EA-treated samples at 1, 3 and 24 hours after stimulation, respectively; NC: negative PCR control containing no cDNA template in the PCR mixture; G3PDH served as a loading control. The following tissues expressed the *Aig1l* gene in (a) and (b): brain, B; muscle, M; heart, H; lung, Lu; spleen, S; liver, L and kidney, K. NC indicates negative control.

All tissues, brain, skeletal muscle, spleen, heart, lung, kidney and liver, expressed the *Aig1l* gene as determined by semiquantitative RT-PCR: the highest expression was found in the brain (data not shown). Northern blot analysis performed in the normal sensitivity mode showed high *Aig1l* gene expression in the brain (Figure 3(a)); the northern blot analysis performed in the high sensitivity mode revealed very weak gene expression in the heart, lung and spleen (data not shown). In addition, real-time quantitative PCR analysis also indicated that the brain had the highest *Aig1l* gene expression and all other tissues had very weak gene expression (Figure 3(b)).

We then analyzed the effect of EA on *Aig1l* expression in skeletal muscle by using semiquantitative RT-PCR. EA induced expression of *Aig1l* at each time point. We found the highest expression after 3 hours of EA (Figure 3(c)).

## 4. Discussion

In this research, we determined the full-length cDNA sequence of the mouse *Aig1l* gene, which EA stimulation induced in mouse skeletal muscle, and analyzed its expression. Because skeletal muscle expressed the *Aig1l* gene after EA stimulation, this gene may be involved in the molecular mechanism of the effectiveness of EA therapy.

We performed full-length cDNA cloning of the *Aig1l* gene, including the transcriptional start site. We determined this site via the cap site hunting method (Figure 1). The complete cDNA sequence was 6073 bp long and mapped to chromosome 5 in the mouse genome database. The mouse *Aig1l* gene is a homologue of the human *SEZ6L* gene [11]. The coding sequences of these genes showed 79% homology. The human *SEZ6L* gene is a homologue of the mouse *Sez6* gene, which research on seizures has detected [12, 13]. The coding sequences for *Aig1l* and the mouse *Sez6* showed 54.8% homology. This finding may reflect the effect of EA stimulation on the brain and nervous system in view of the relation among the gene *Aig1l* and the seizure-related genes *Sez6* and *SEZ6L*.

Our investigation of the tissue distribution of *Aig1l* gene expression revealed the greatest expression in the brain, as evidenced by semiquantitative RT-PCR (data not shown), northern blot analysis (Figure 3(a)) and real-time quantitative PCR analysis (Figure 3(b)). All other tissues evaluated—skeletal muscle, heart, lung, spleen, liver and kidney—had much weaker *Aig1l* expression than the brain (Figures 3(a) and 3(b)). The human *SEZ6L* gene was also reportedly detected in various tissues [11].

Bioinformatics analysis of *Aig1l* near the transcriptional control region showed Sp1, CP2 and MZF1 binding motifs. Sp1 and CP2 are ubiquitously expressed transcription factors. Sp1 functions in development and differentiation [14, 15], and CP2 operates in regulation of erythroid genes [16, 17]. MZF1 is involved in proliferation and differentiation [18]. These bioinformatics results agree with findings on the tissue distribution of *Aig1l* gene expression.

Our analysis with the Pfam database showed that *Aig1l* has CUB and Sushi domains (Figure 2). CUB domains occur almost exclusively in extracellular and plasma membrane-associated proteins, and in proteases [19–21]. Those proteins reportedly participate in a wide range of biological functions: developmental processes [22–25], neurotransmission [26], cell signaling [27], hemostasis [20], tumor suppression [28], inflammation [29] and complement activation [21]. Sushi domains are known as complement control protein modules, or short consensus repeats, found in complement and adhesion proteins [30]. Sushi domain-containing proteins operate in regulation of the complement system and in blood clotting. Involvement of these proteins in cell adhesion [30], embryogenesis [31] and blood coagulation [32] has also been reported. In addition, the human *SEZ6L* and mouse *Sez6* genes, the homologous genes of *Aig1l*, have both CUB and Sushi domains [11–13]. The *Sez6* gene is reportedly important for neuronal information transfer, which Gunnersen et al. [33] determined on the basis of analysis using *Sez6* knockout mice. Their report showed that *Sez6* proteins are important for normal dendritic arborization of cortical neurons and for development of appropriate excitatory synaptic connectivity. All these reports suggest that *Aig1l* may be associated with neurotransmission and protein-protein interaction. In view of the relation between those domains of *Aig1l* and the effect of EA, further analysis of *Aig1l* focused on neurotransmission and protein-protein interaction may reveal the functional importance of this gene.

In addition, we examined chronological changes in *Aig1l* gene expression to verify the effect of EA stimulation of skeletal muscle on gene expression. We found that EA did induce *Aig1l* gene expression, with the highest expression measured after 3 hours of EA (Figure 3(c)). This result suggests that the *Aig1l* gene is related to the rapid effect of EA stimulation.

During the process of *Aig1l* gene detection, we utilized transcriptome and histochemical analyses and discovered that EA induced a satellite cell-related proliferative reaction in skeletal muscle [2]. Certain other research groups have also reported transcriptome analysis of EA, which revealed the efficacy of EA stimulation: Kim et al. [5] showed that EA increased natural killer cell activity with high efficacy, and Ko et al. [6], by using cDNA microarray analysis, reported that the opioid receptor was involved in analgesic processes of EA. Thus, transcriptome analysis may be one of the most effective approaches for research in complementary and alternative medicine.

In conclusion, we determined the full-length cDNA sequence of the *Aig1l* gene and its expression in various tissues (mainly brain). We showed chronological changes in gene expression, with the greatest expression induced after 3 hours of EA. However, the biochemical function of the *Aig1l* gene is not as yet fully explained. Additional investigations of the *Aig1l* gene, including the relation between this gene and EA, are warranted.

## Funding

20th Grant-in-aid of the Nakatomi Foundation in the fiscal year 2007.
